# Supplementary material for: Using Triethylborane to Manipulate Reactivity Ratios in Epoxide-Anhydride Copolymerization: Application to the Synthesis of Polyethers with Degradable Ester Functions
Source: Molecules. 2022 Jan 11;27(2):466. doi: 10.3390/molecules27020466 (PMC8780197; doi:10.3390/molecules27020466)
Supplement: Supplementary file 1 [file molecules-27-00466-s001.zip › molecules-1535508-supplementary.pdf]

Using Triethylborane to Manipulate Reactivity Ratios in Epoxide-Anhydride Copolymerization: Application to the Synthesis of Polyethers with Degradable Ester Functions

Vamshi K. Chidara, Yves Gnanou* and Xiaoshuang Feng*

Physical Sciences and Engineering Division, King Abdullah University of Science and Technology (KAUST), Thuwal-23955, Saudi Arabia; vamshi.chidara@kaust.edu.sa (V.C.)

* Correspondence: yves.gnanou@kaust.edu.sa (Y.G.); fxs101@gmail.com (X.F.)

SUPPLEMENTARY MATERIAL

Table of Content

Figure S1. Representative *in situ* ^{1}H NMR kinetic experiment and Jaacks plot for entry 3, Table 1 .... **2**

Figure S2. Representative *in situ* ^{1}H NMR kinetic experiment and Jaacks plot for entry 4, Table 1 .... **3**

Figure S3. Representative *in situ* ^{1}H NMR kinetic experiment and Jaacks plot for entry 5, Table 1 .... **4**

Figure S4. Representative *in situ* ^{1}H NMR kinetic experiment and Jaacks plot for entry 6, Table 1 .... **5**

Figure S5. Representative *in situ* ^{1}H NMR kinetic experiment and Jaacks plot for entry 7, Table 1 .... **6**

Figure S6. Representative *in situ* ^{1}H NMR kinetic experiment and Jaacks plot for entry 8, Table 1 .... **7**

Figure S7. Representative GPC traces for SA/EO copolymers in entries 1,2,4 in Table 2.......................... **8**

Figure S8. Representative GPC traces for PA/EO copolymers in entries 5,6,7 in Table 2.......................... **9**

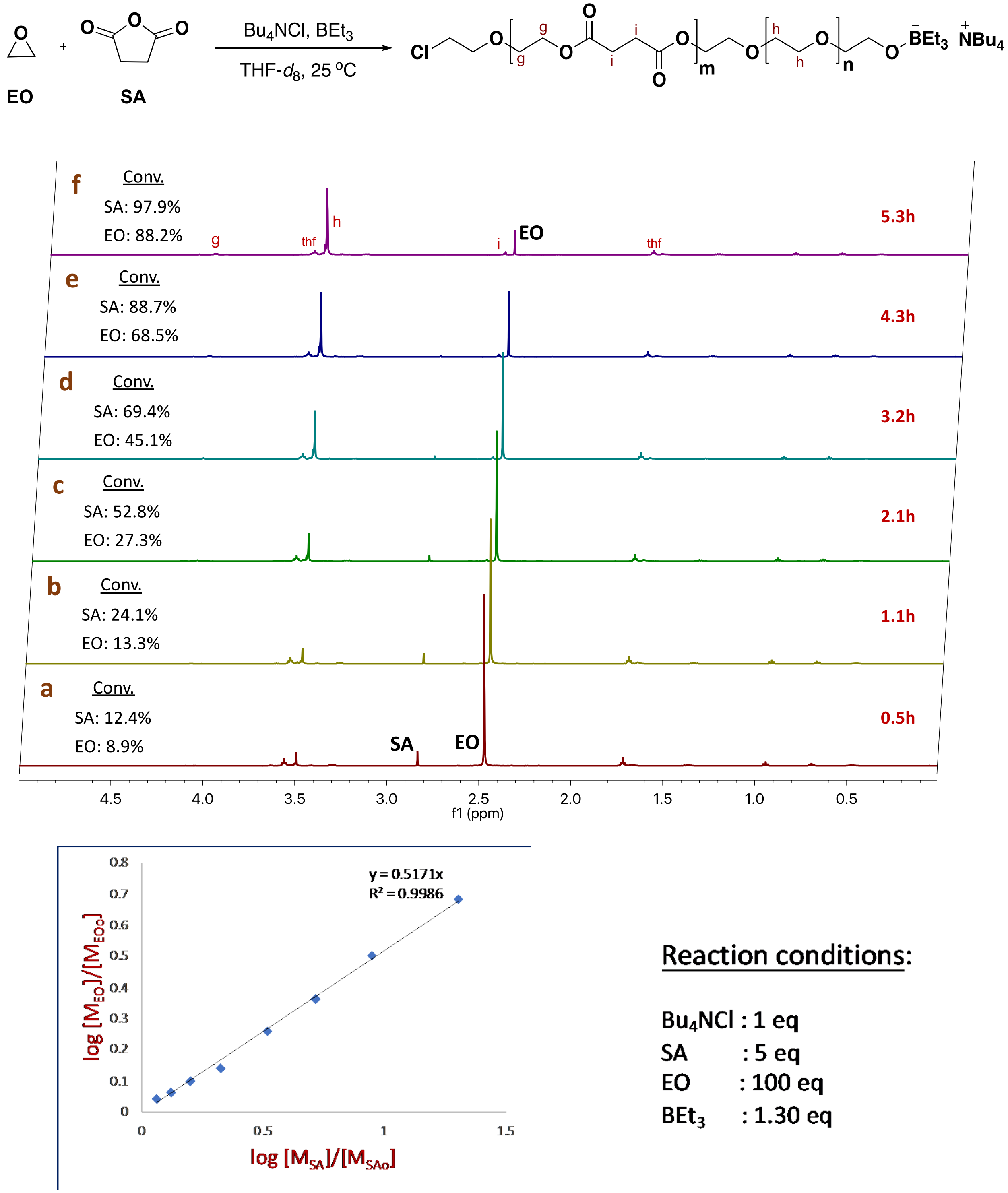

Figure S1. Representative *in situ* ^{1}H NMR kinetic experiment and Jaacks plot for entry 3, Table 1

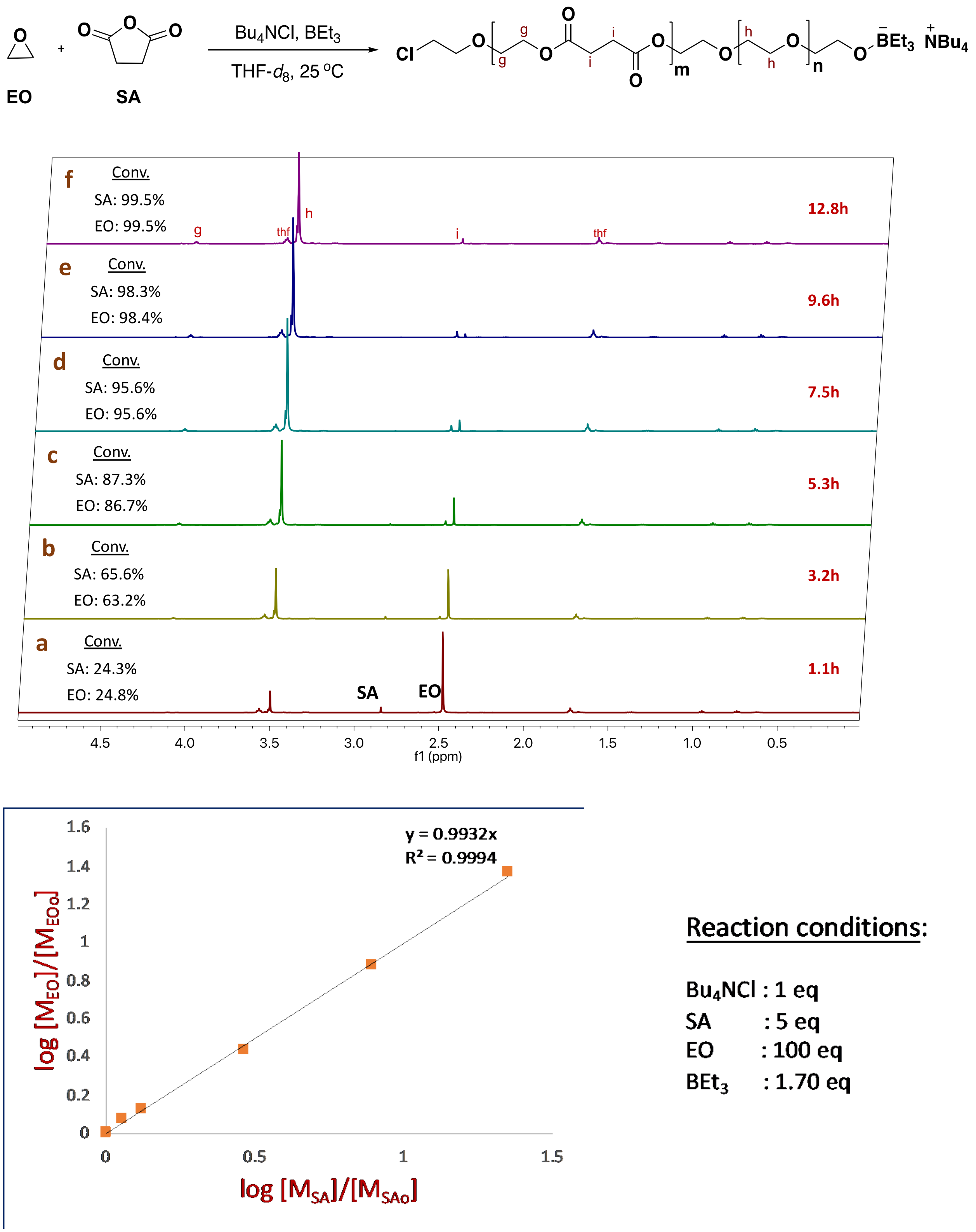

Figure S2. Representative *in situ* ^{1}H NMR kinetic experiment and Jaacks plot for entry 4, Table 1

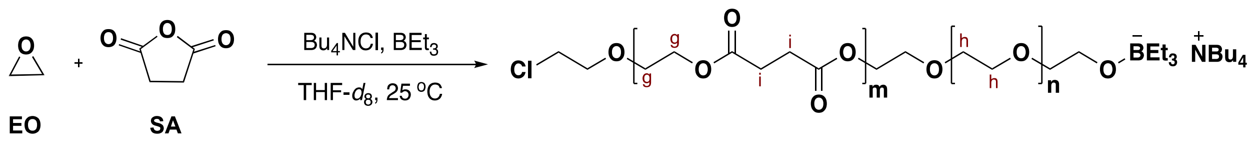

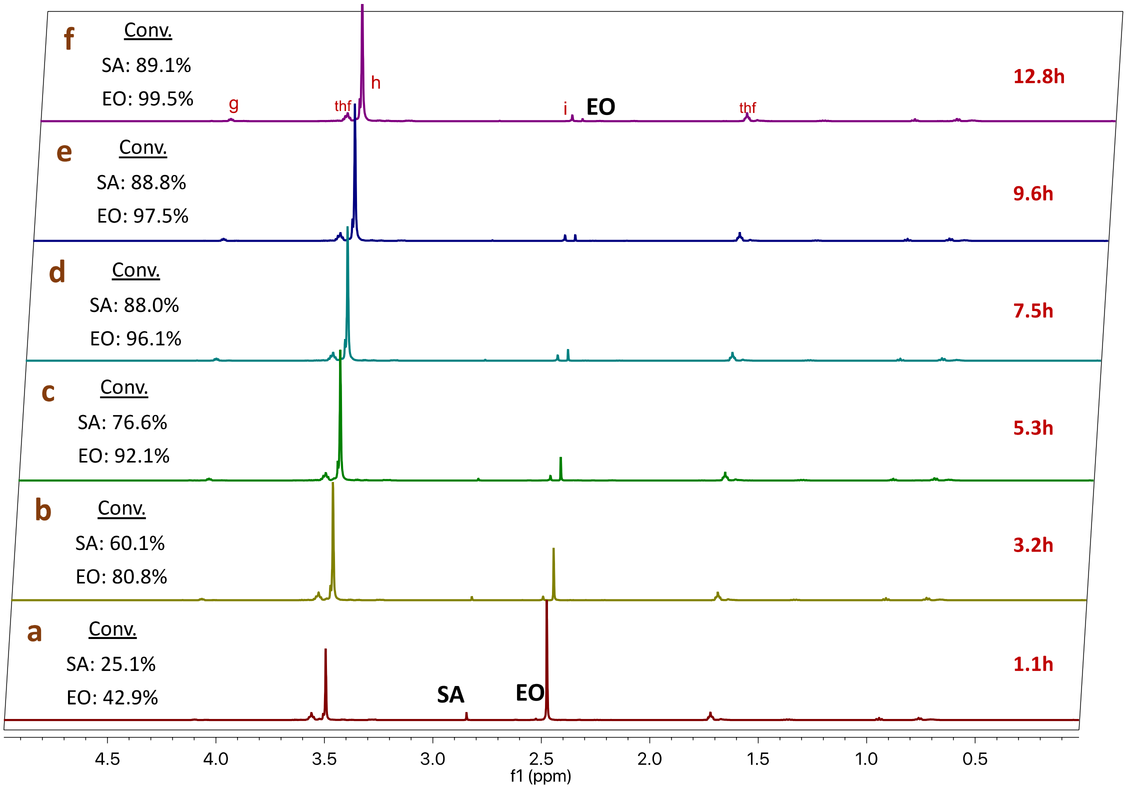

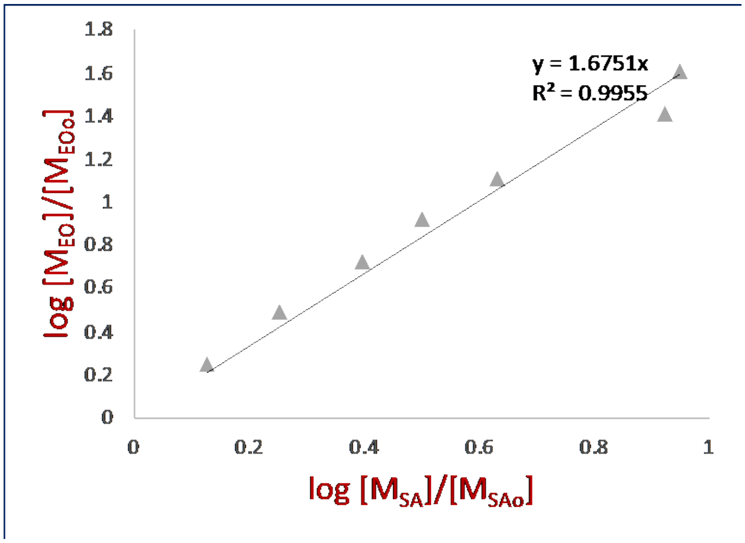

Reaction conditions:

Bu_4NCl	: 1 eq
SA	: 5 eq
EO	: 100 eq
BEt_3	: 2.30 eq

Figure S3. Representative *in situ* ^{1}H NMR kinetic experiment and Jaacks plot for entry 5, Table 1

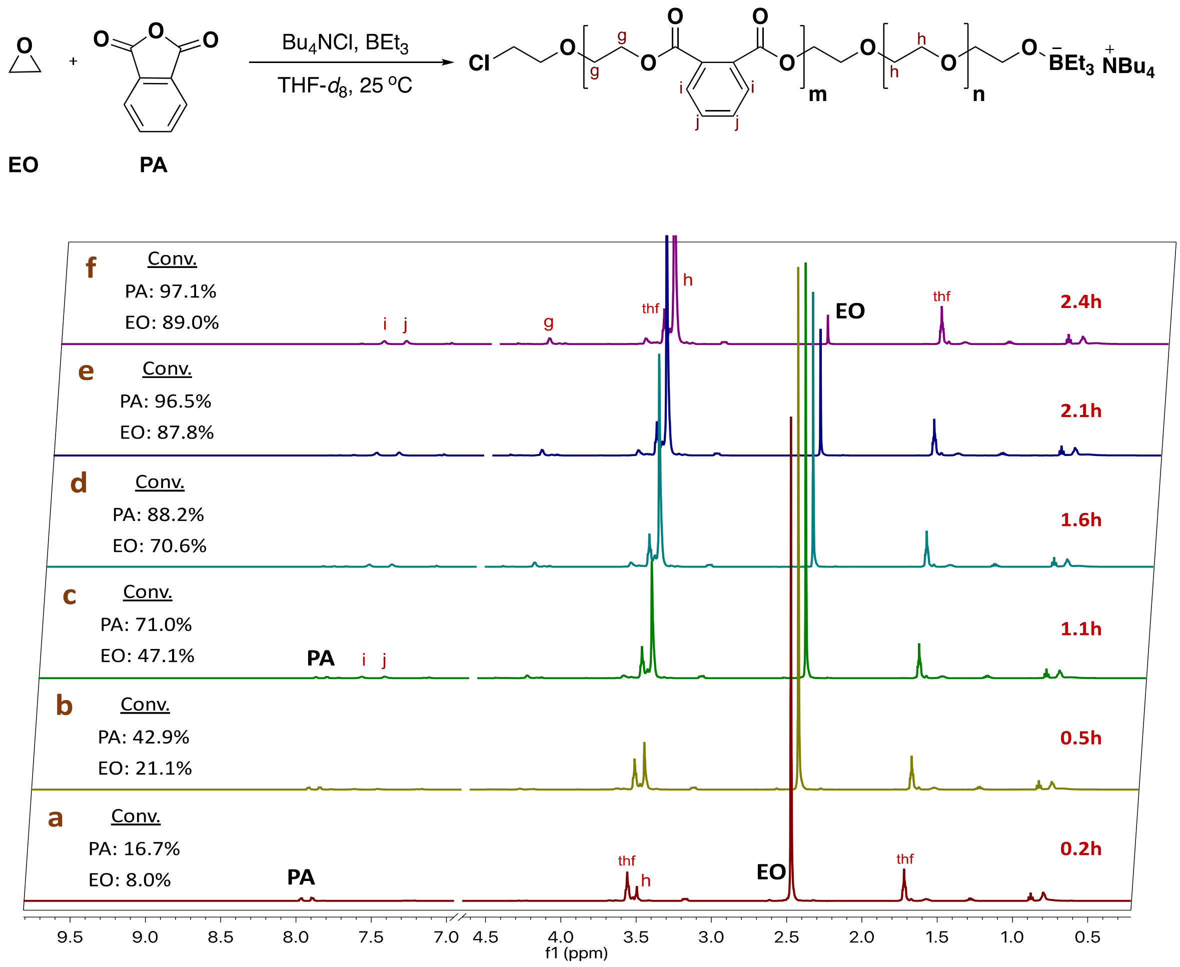

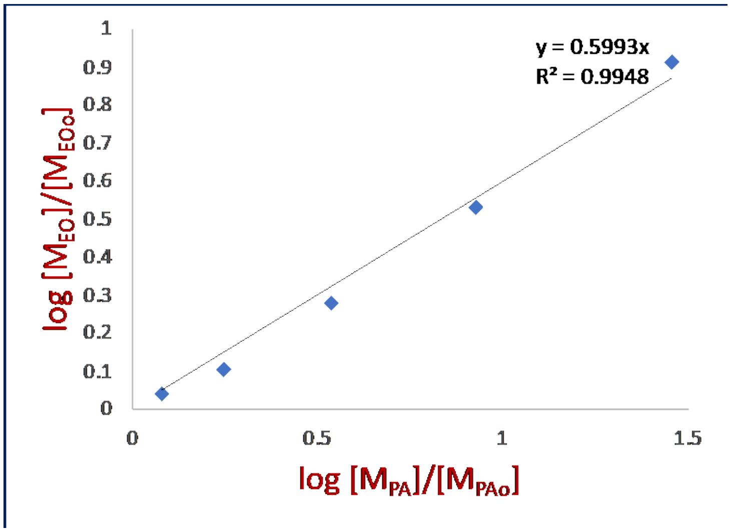

Reaction conditions:

Bu_4NCl : 1 eq
PA : 5 eq
EO : 100 eq
BEt_3 : 2.3 eq

Figure S4. Representative *in situ* ^{1}H NMR kinetic experiment and Jaacks plot for entry 6, Table 1

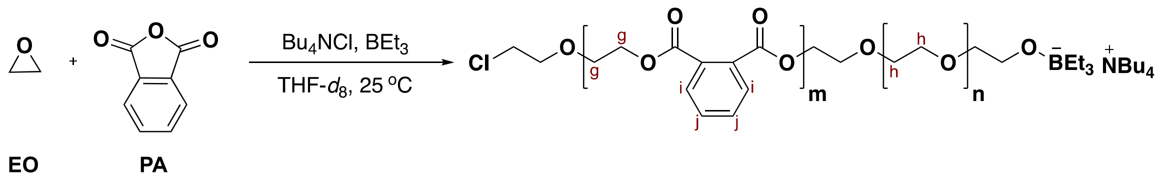

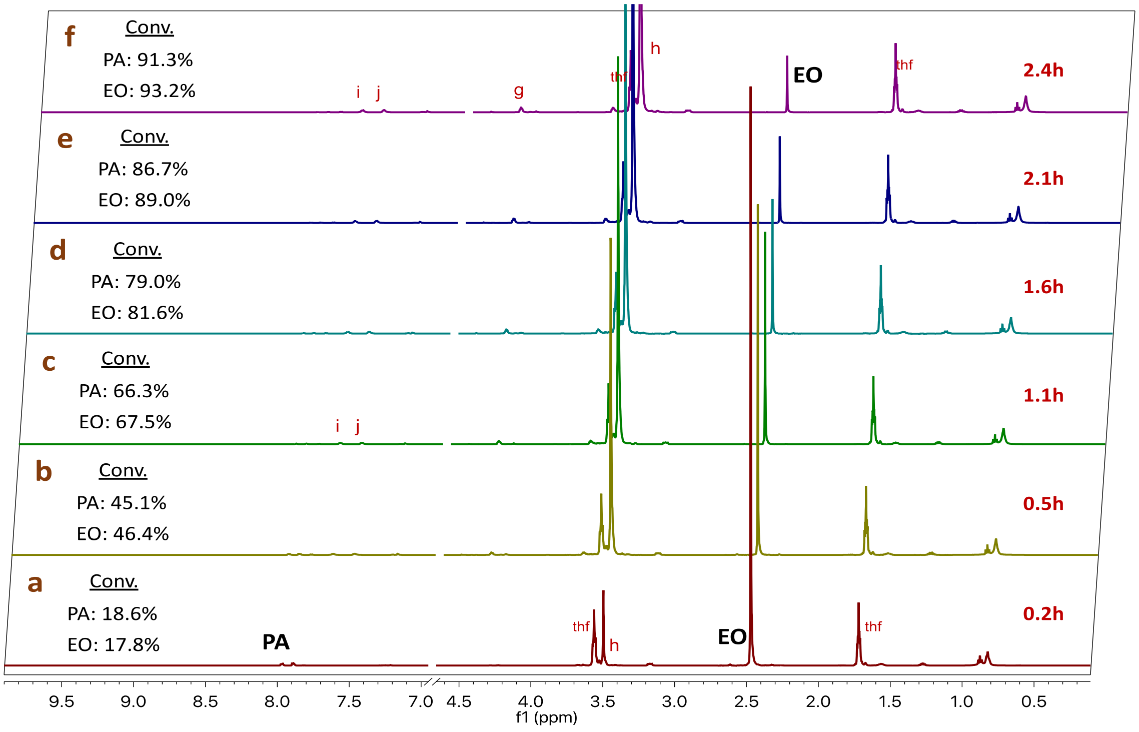

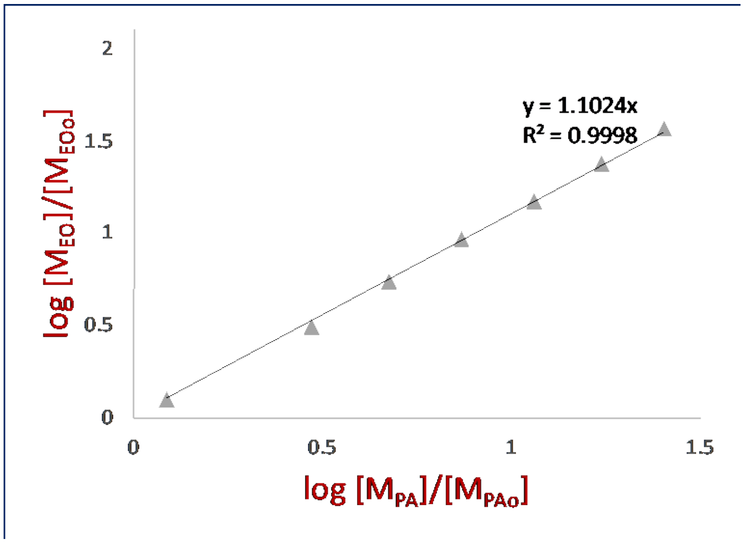

Reaction conditions:

Bu_4NCl : 1 eq
PA : 5 eq
EO : 100 eq
BEt_3 : 3.8 eq

Figure S5. Representative *in situ* ^{1}H NMR kinetic experiment and Jaacks plot for entry 7, Table 1

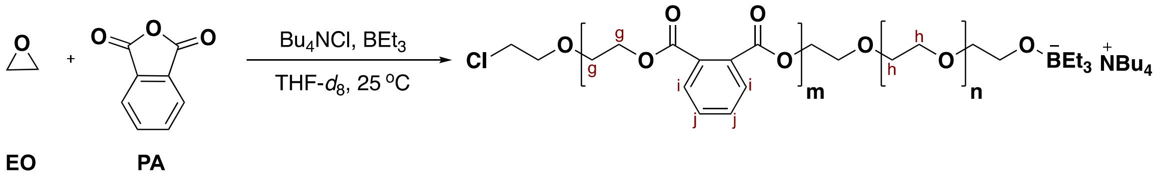

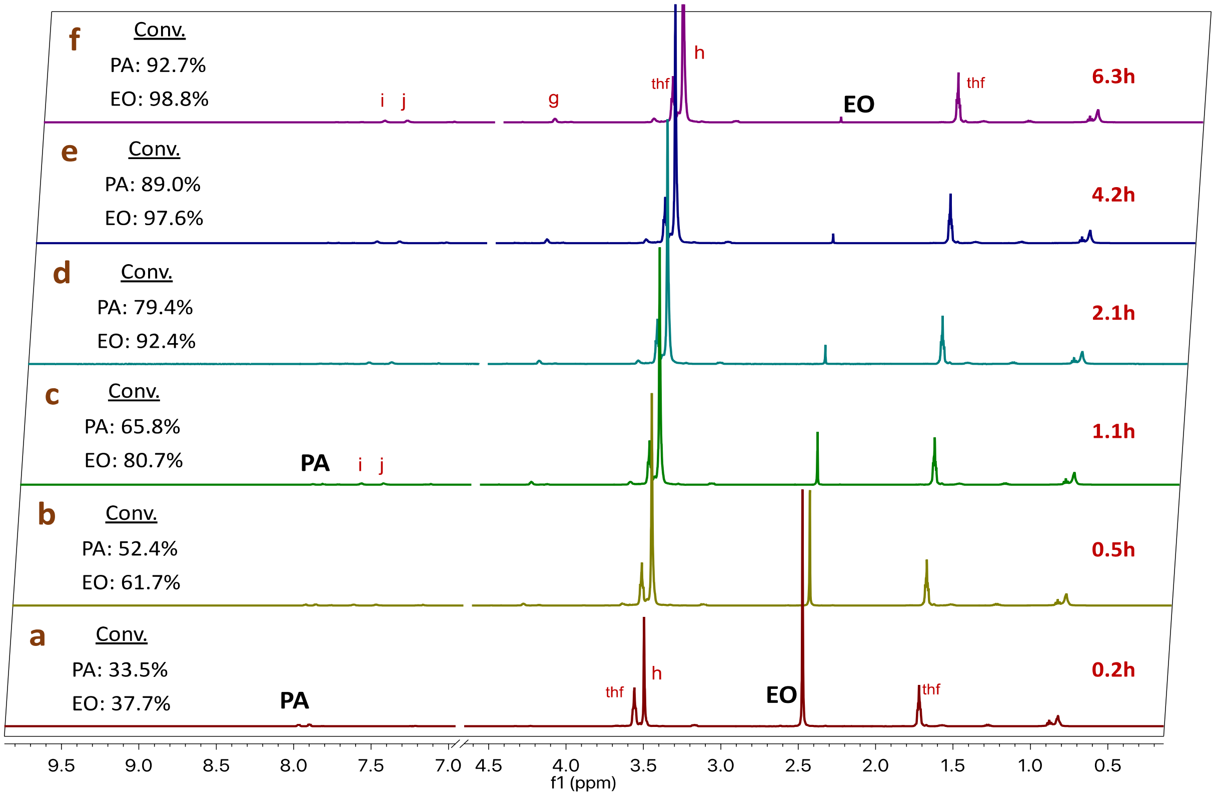

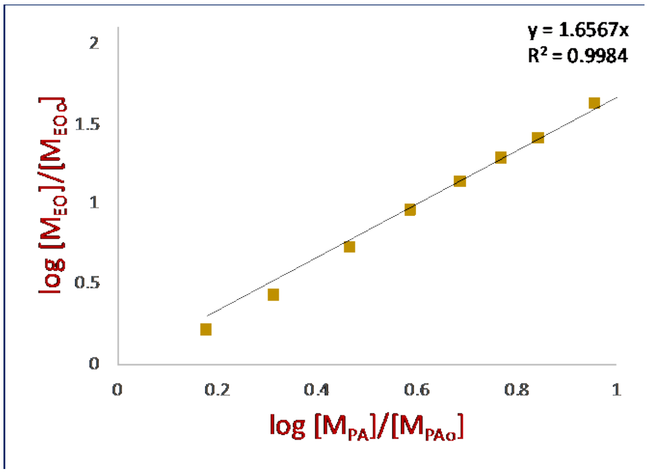

Reaction conditions:

Bu_4NCl : 1 eq
PA : 5 eq
EO : 100 eq
BEt_3 : 4.1 eq

Figure S6. Representative *in situ* ^{1}H NMR kinetic experiment and Jaacks plot for entry 8, Table 1

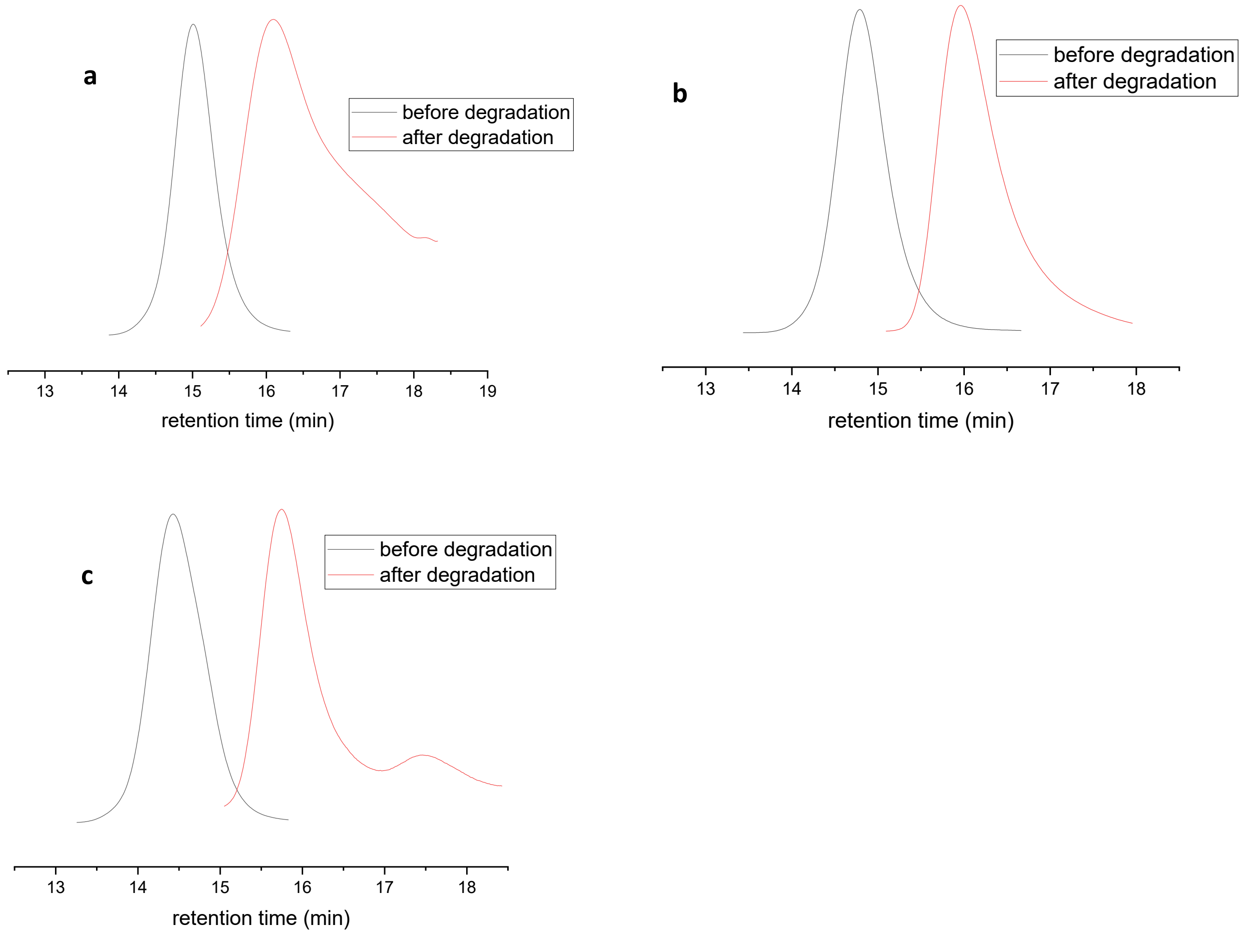

Figure S7. Representative GPC traces for SA/EO copolymers in entries 1,2,4 in Table 2. Black trace represents molar mass of polymer sample before degradation and red trace is after degradation. a: entry 1; b: entry 2; c: entry 4.

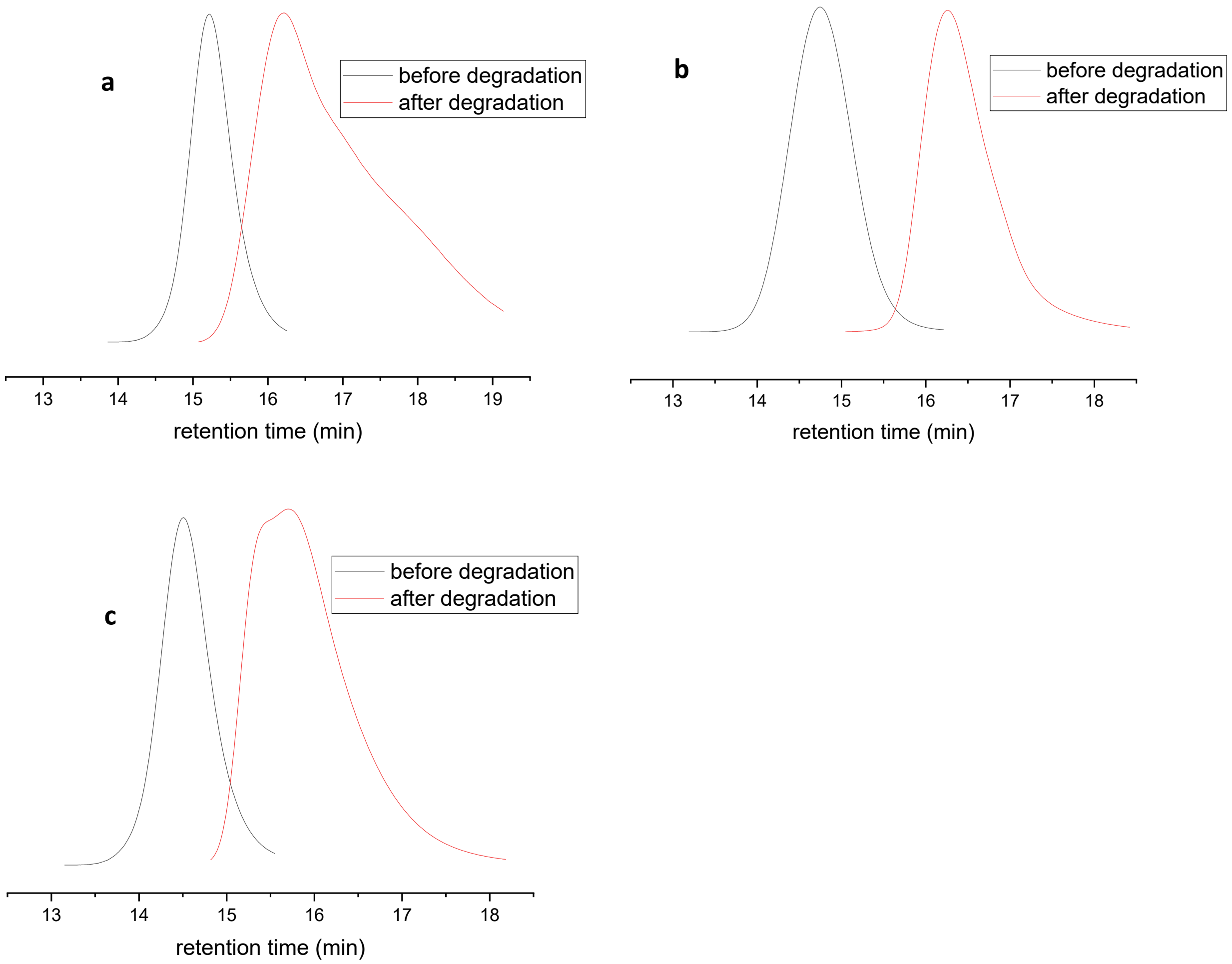

Figure S8. Representative GPC traces for PA/EO copolymers in entries 5,6,7 in Table 2. Black trace represents molar mass of polymer sample before degradation and red trace is after degradation. a: entry 5; b: entry 6; c: entry 7.
